# Acute and Chronic Effect of Acoustic and Visual Cues on Gait Training in Parkinson's Disease: A Randomized, Controlled Study

**DOI:** 10.1155/2015/978590

**Published:** 2015-11-26

**Authors:** Roberto De Icco, Cristina Tassorelli, Eliana Berra, Monica Bolla, Claudio Pacchetti, Giorgio Sandrini

**Affiliations:** ^1^C. Mondino National Neurological Institute, 27100 Pavia, Italy; ^2^Department of Brain and Behavioral Sciences, University of Pavia, 27100 Pavia, Italy

## Abstract

In this randomized controlled study we analyse and compare the acute and chronic effects of visual and acoustic cues on gait performance in Parkinson's Disease (PD). We enrolled 46 patients with idiopathic PD who were assigned to 3 different modalities of gait training: (1) use of acoustic cues, (2) use of visual cues, or (3) overground training without cues. All patients were tested with kinematic analysis of gait at baseline (T0), at the end of the 4-week rehabilitation programme (T1), and 3 months later (T2). Regarding the acute effect, acoustic cues increased stride length and stride duration, while visual cues reduced the number of strides and normalized the stride/stance distribution but also reduced gait speed. As regards the chronic effect of cues, we recorded an improvement in some gait parameters in all 3 groups of patients: all 3 types of training improved gait speed; visual cues also normalized the stance/swing ratio, acoustic cues reduced the number of strides and increased stride length, and overground training improved stride length. The changes were not retained at T2 in any of the experimental groups. Our findings support and characterize the usefulness of cueing strategies in the rehabilitation of gait in PD.

## 1. Introduction

Parkinson's disease (PD) is a degenerative neurologic disorder characterized by motor and nonmotor symptoms. Gait disorders are a hallmark of idiopathic PD and several studies have highlighted a typical parkinsonian walking pattern characterized by reduced speed, increased duration of the stance phase, shorter stride length, and increased number of strides [1, 2]. Although many symptoms respond well to antiparkinsonian drugs, gait and balance impairment often show a poor response to pharmacological treatment. In this frame, physical therapy acquires an important role in contributing to the management of this kind of symptoms. Advanced rehabilitation techniques have been proposed over the years: these include treadmill walking [3], direct current stimulation [4], and ground training with cues [5]. Cues are defined as external stimuli of different type, that is, instructional, auditory, visual, and sensory, and are applied to improve gait performance via the activation of different strategies of motor control. Auditory cues, for instance, are believed to provide an external rhythm that bypasses internal rhythm deficit [6] and visual cues engage the visual-cerebellar motor pathway to facilitate the generation of a better gait pattern [7], whereas sensory cues enable the voluntary activation of the dorsolateral premotor control system, thus bypassing the failure of supplementary motor area in controlling automatic movement [8, 9].

Several studies show that the use of external cues is effective in improving gait parameters [5]. However only a few of these studies are randomized controlled trials and virtually none of them has compared the chronic effect of different external cues. In our practice, we have noted that some patients tend to respond better to a specific type of cue, which prompts the idea that cues may have a different profile of effect.

Rehabilitation of gait is progressively becoming a mainstay in the management of advanced phases of PD. Several approaches have been proposed in recent years, including individual or group rehabilitation in the outpatient setting and home-based therapy [10, 11]. In general, these studies show that home exercises are less effective in improving balance, gait, and functional measures and that home-based therapy is associated with lower compliance and higher complication rates (i.e., falls or muscle-tendon injuries), especially in patients with balance impairment or other medical complications [12–14]. Frazzitta et al. have shown the effectiveness of a combined gait training modality based on visual or auditory cues, associated or not with treadmill device, delivered to inpatients over a period of 4 weeks [15].

The aim of the present study was the comparison and the characterization of the acute and chronic effects of visual and acoustic cues, used individually, in gait rehabilitation of PD. The study was conducted on PD patients hospitalized for neurorehabilitation at our Unit and was designed as a randomized controlled study for parallel groups, where patients were assigned randomly to one of the following groups for gait training: (1) use of acoustic cues (rhythmical sounds), (2) use of visual cues (stripes of contrasting colour), or (3) overground training without any cues. The objective of the study was to quantify the changes induced by the 3 different approaches applied for 4 weeks in an intensive rehabilitative programme on (i) gait parameters, measured by means of the kinematic analysis of gait, and (ii) the clinical picture, measured by means of the Unified Parkinson's Disease Rating Scale (UPDRS) and the Functional Independence Measure (FIM).

## 2. Materials and Methods

### 2.1. Subjects

The subjects were enrolled among consecutive PD patients hospitalized in the Neuro-Rehabilitation Unit of the C. Mondino National Neurological Institute of Pavia, Italy. Hospitalization for neurorehabilitation is a routine procedure at our Institute, as we know from our long-time experience and from data from the literature that inpatient-delivered rehabilitation, with strictly supervised physical therapy, is associated with a greater benefit in patients affected by PD with moderate-severe degrees of motor impairment [16, 17]. We also know from our clinical experience that, for a correct use of cues, at least for the initial sessions, patients need clear instructions and supervision from the therapist. Taking into consideration all these conditions, we opted for an inpatient setting for our trial to limit bias caused by poor compliance or by cues misuse.

Forty-six patients (24 males, 22 females; age 74.4 ± 7.1 years) affected by Idiopathic Parkinson's Disease, according to the UK Brain Bank diagnostic criteria, were included in this randomized, controlled, parallel-group study. Patients were hospitalized upon referral of a neurologist trained in Movement Disorders, who visited the patients in the outpatient clinic and prescribed rehabilitation for any or a combination of the following conditions: decline in global motor performances, increase in the risk of falls, marked reduction of walking endurance, or worsening of bradykinesia.

Inclusion criteria were Hoehn and Yahr stage between II and IV, MMSE > 23, and no changes in antiparkinsonian drug treatment in the previous 6 months. Exclusion criteria were positive history for neoplasms, cardiovascular disease, respiratory disease, clinically significant muscular-skeletal disease, other neurological conditions, uncorrected visual or auditory disturbances, or hospitalization in the previous 3 months.

Patients were divided into 3 groups who were randomly assigned to three different treatment approaches for gait training (with a 1 : 1 : 2 ratio): walking in the presence of rhythmical sounds (Acoustic Group, *n* = 11), walking on stripes of contrasting colour with respect to the floor (Visual Group, *n* = 11), and overground training without cues (Control Group, *n* = 24).

### 2.2. Cueing Strategies and Rehabilitative Intervention

Patients in all the 3 groups underwent 5 daily rehabilitation sessions per week for 4 consecutive weeks. These sessions consisted in 40 min treatment with passive muscle stretching, exercises for rigidity and joint mobility, specific motor exercise for hypokinesia, weight shifting, and balance training for posture and movement strategies to prevent falls. In addition, patients underwent 5 daily sessions per week for 4 weeks dedicated to gait training as described below. Each session lasted 20 minutes.

In the Acoustic Group, cues consisted in a rhythmical digital sound (“beep”) emitted by a digital metronome, with a frequency ranging between 60 and 120 Hz. The beep cadence was personalized and optimized for each patient during the first rehabilitative session by the physical therapist.

In the Visual Group, cues consisted in coloured stripes placed on the floor perpendicularly to the walking direction. The interstripe distance was personalized and optimized by the physical therapist during the first rehabilitative session. The physical therapist tested each subject with different distances between the stripes, starting from a minimum distance of 25 cm to a maximum of 60 cm. The therapist asked the patient to walk over the stripes trying to step over the next stripe and avoiding trampling on them.

In the Control Group, gait training was performed overground, without the use of any cue.

### 2.3. Study Design and Protocol

All patients were examined by a neurologist with expertise in Movement Disorder at the beginning of hospitalization (T0), at the end of the neurorehabilitation period (+4 weeks, T1), and 3 months after discharge from the hospital (T2). At each time point, the patients were tested with the Unified Parkinson's Disease Rating Scale, motor part (UPDRS-III) [18] and with the Functional Independence Measure (FIM) [19].

For the evaluation of the* chronic effect* of the 3 types of gait rehabilitation, the kinematic analysis of gait was recorded a T0, T1, and T2 in uncued condition in all 3 experimental groups. The* acute effect of cues* was evaluated at T0 in the Acoustic and Visual Groups by recording gait during conditioning with the visual or the auditory cue.

All patients enrolled in the study were tested in the morning, always in the ON condition.

Antiparkinsonian drugs schedule was kept steady for the entire study duration.

### 2.4. Kinematic Analysis of Gait

Kinematic analysis of gait was performed with a 6-camera optoelectronic system (ELITE, BTS Engineering, Milan, Italy) by an experienced laboratory technician with a sampling rate of 100 Hz. Twenty-one spherical reflective markers (15 mm in diameter) were applied along the body according to the Davis protocol [20]. Synchronized acquisition and data processing were performed using the Analyzer software (BTS, Milan, Italy). In order to perform kinematic analysis of gait, patients were instructed to walk at their normal speed along a 7-meter walkway. For every session, at least four gaits per patient were recorded and analysed.

We collected the following variables: number of strides needed to walk 7 meters, speed of gait, stride duration and stride length, percentage duration of swing and stance phases.

### 2.5. Ethics Approval

The local Ethics Committee approved the study protocol and all the participants gave their written informed consent before enrolment.

### 2.6. Power Analysis

We considered as our primary outcome measure the chronic effect of gait rehabilitation with cues on the number of strides at the end of the 4-week rehabilitation period. We knew from our clinical experience that patients with PD employed an average of 6-7 strides to walk the 7-meter walkway of our laboratory. Based on our practice and on data from the literature we considered as clinically meaningful a difference between groups after rehabilitation greater than one stride, which corresponds to a difference of at least 20% between groups [21].

Therefore, we calculated the sample size with the following parameters: confidence interval (two sided) 95%; power 80%; difference between groups 20% (with a standard deviation between 20 and 25% for each group). The suggested sample size was of 42 patients. We planned to enlarge the study group of a further 10% considering possible drop-outs, so we decided to enroll 46 patients, to be distributed into the 3 different arms.

### 2.7. Statistical Analysis

The Statistical Package for the Social Sciences (SPSS) for Windows, version 21.0, was used for the calculation.

For each variable we evaluated “skewness” and “kurtosis” to assess normality. Moreover the data were plotted using a “*Q*-*Q* plot” that confirmed normal distribution of all tested variables. For qualitative variables we used cross-tabs analysis, performing statistical significance with chi-square or Fisher exact test by case. Quantitative variables are presented as mean values ± standard deviation.

Regarding the* acute effect* of cues on gait parameters, we performed an intragroup analysis comparing data recorded with and without cues walking using Student's* t*-test for paired groups. For the purpose of our study, we did not assess intergroup differences for acute effects.

Regarding the* chronic effect of the different modalities of gait training*, we performed both an intragroup and an intergroup analysis. To assess intragroup effects in presence of multiple time measurements (T0 versus T1 versus T2), we performed an ANOVA (analysis of variance) test for repeated measures, with post hoc Bonferroni's correction, for each study group. To assess differences between groups, at each time point, we used an ANOVA test for multiple unpaired groups, with Bonferroni's post hoc. The level of significance (*α*) was set for convention as *p* < 0.05, always corrected if necessary.

## 3. Results

Demographic and clinical characteristics of the 3 groups are shown in Table 1. The table also shows the baseline gait parameters for the 3 groups under investigation. No statistically significant differences were found between groups.

### 3.1. Acute Effect of Cues on Gait Parameters

Use of acoustic cues induced a significant increase in stride duration and in stride length (Table 2). Visual cues caused a decrease in the number of strides, an increase in the percentage of time spent in the swing phase with a corresponding reduction in the time spent in the stance phase, and a reduction in the gait speed (Table 3).

### 3.2. Chronic Effect of the 3 Types of Gait Training

#### 3.2.1. Gait Parameters

At the end of the 4-week rehabilitation programme, in the Acoustic Group we observed a significant decrease in the number of strides, an improvement in stride length, and an increase in the speed of gait (Table 4).

In the Visual Group we found a significant reduction in the number of strides, an increase in the speed of gait, and an increase in the duration of the swing phase with a corresponding reduction in the stance phase. At T1 the reduction in the number of strides was associated with an increase in stride length, which however did not reach a statistical significance (Table 5).

In the Control Group we detected an increase in stride length and in gait velocity (Table 6).

**Figure 1:**
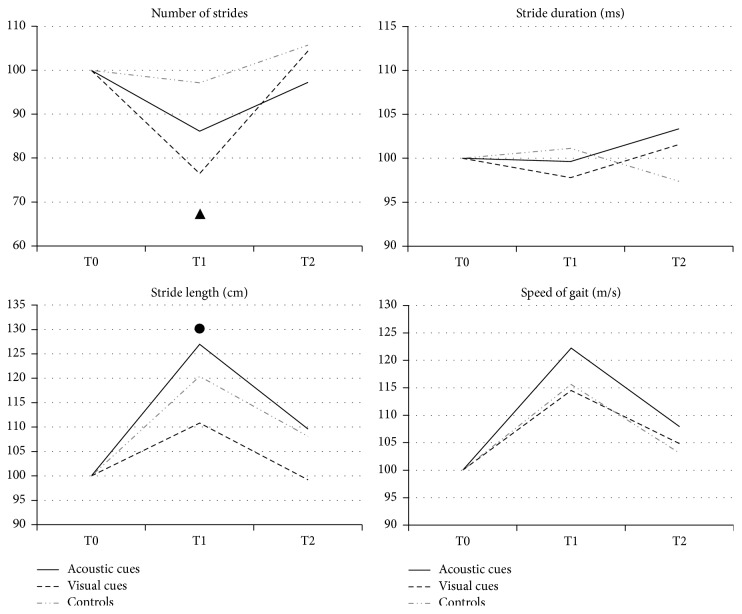
Effect of the different modalities of gait training on gait parameters recorded by means of the kinematic analysis. Baseline values are normalized to 100% and changes represented as % variation from baseline. ▲ Acoustic Group versus Controls *p* < 0.05 and Visual Group versus Controls *p* < 0.05. ● Acoustic Group versus Visual Group *p* < 0.05 and Controls versus Visual Group *p* < 0.05.

When comparing the 3 groups (Figure 1), at T1 we found that the number of strides was significantly lower in both groups treated with cues (Acoustic and Visual) with respect to Controls, while the stride length increased significantly more in the Acoustic Group and in the Control Group than in the Visual Group. In all the three groups of patients, the improvement in gait parameters was lost at the 3-month follow-up (T2).

**Figure 2:**
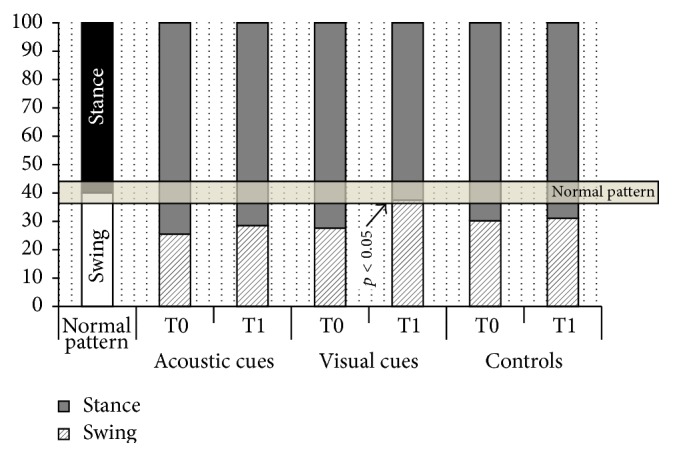
Distribution of stance and swing phases in the 3 treatment groups at T0 and T1. The first column on the left shows the normal percent distribution of the 2 phases of gait. The shaded horizontal bar represents the normal variability of gait pattern in healthy subjects (±4%). Note that parkinsonian gait is characterized by a reduction in the swing phase and that visual cues normalized the distribution of these 2 phases at T1. Visual Group: T0 versus T1 *p* < 0.05. At T1 Visual Group versus Acoustic Group *p* < 0.05 and Visual Group versus Controls *p* < 0.05.

Interestingly, at T1 in the Visual Group we observed a significant increase in the time spent during the swing phase (with a corresponding decrease in the stance phase). This redistribution normalized the gait pattern of the patients, bringing the swing/stance ratio within the normal variability range in this treatment group (Figure 2).

### 3.3. UPDRS and FIM Scales

UPDRS-III significantly decreased at T1 in all the 3 groups under evaluation, whereas at T2 the improvement in UPDRS-III was no longer detectable.

FIM significantly improved at T1 in all groups of patients, but the gain was not preserved at T2. No statistically significant differences were found between groups at any time point in neither scale (Table 7).

## 4. Discussion

In the last years rehabilitation has assumed a growing importance as part of a multidisciplinary approach to PD. One of the most affected motor tasks in PD is gait, due to a deficit of internal rhythmic signals, which interferes in motor performance [7].

Data from the literature show that external stimuli (acoustic, visual, and somatosensory) are able to modulate the motor pattern in PD, helping the patients to start and maintain a rhythmic motor task [8]. Cued gait training seems to represent a precious aid for managing PD symptoms not (or not any longer) responding to dopaminergic drugs, as cues seem to be able to access rhythmic entrainment mechanisms also in the absence of dopaminergic stimulation. Indeed McIntosh et al. [6] studied the effect of acute rhythmic auditory stimulation (RAS) in patients with idiopathic Parkinson's disease also during the OFF phase and reported an improvement in the majority of patients. Cues may also be effective in freezing, a severe gait disturbance that responds poorly to dopaminergic stimulation [6]. Arias and Cudeiro [22] investigated the acute effect of RAS on the gait of PD patients with and without freezing of gait during the end-of-dose periods. The authors report a significant reduction in the number and duration of freezing episodes under RAS conditioning, with a reduction in the time to turn and an increase in cadence and velocity in both groups of patients, with and without freezing.

Most of the randomized controlled trials aimed at evaluating the effect of auditory and visual cues on gait in PD have focused on the immediate effect on gait of the cues [23–41]. Some other studies evaluated the chronic effect (generalization) of auditory and visual cue, individually [42, 43] or used in combination [15, 44, 45]. In general, this wealth of studies showed that both auditory cueing and acoustic cueing are effective in improving some parameters of walking. Auditory cueing seems more effective on speed, cadence, and step length, whereas visual cues ameliorate speed cadence and step length [5, 46]. A limited number of studies have evaluated the retention of the beneficial effect, once the rehabilitation has been stopped [42, 43, 45, 47, 48]. The duration of follow-ups ranges from 4 to 8 weeks, and findings are quite controversial. In general, the improvement in gait parameters induced by visual or auditory cues is maintained at the shortest revaluations, but it progressively wanes when the follow-up duration stretches beyond 2 months.

To the best of our knowledge, no randomized controlled trial has analyzed comparatively the acute and chronic effect of the 2 types of cues used individually. An attempt to indirectly compare the efficacy of the two cues on gait parameters was made by Spaulding in the meta-analysis of 2002, where he concluded that auditory cues provided a more consistent and positive effect on gait parameters of PD patients when compared to visual cueing [46]. This aspect seems important, since the different types of cues are believed to engage anatomic pathways with a differential modality [6–8] and, in our practice, we have noticed that patients may respond preferentially to one type of cue, some showing more marked improvement with visual cues, others with auditory cues.

In the present study we investigated the effect of visual or auditory cues upon gait parameters both acutely (walking under cueing) and chronically (walking without cueing after a four-week rehabilitation program). We also investigated whether there was any retention of the effect at 3 months.

Regarding the acute effect, we found a significant increase in stride duration and in stride length when patients were exposed to acoustic cues, while a decrease in the number of strides and a reduction in gait speed were observed in patients exposed to visual cues. The worsening of some features of gait, such as the increase of stride duration with the acute acoustic cue and the reduction of speed with the acute visual cue, was not totally surprising because we realized that a proper use of cues by PD patients requires supervision by the therapist and a learning process by the patient to integrate the cue in the automaticity of gait. At the time of acute evaluation the patients had met the therapist only once and they were not familiar with cues. This observation seems relevant for the practical approach to gait training with cues, because it suggests that the adoption of the cueing strategy in the home-unassisted rehabilitation requires an adequate assisted training to avoid that the patients fail to internalize the cues aid or do so with a less functional motor pattern.

At the end of the rehabilitation program, the patients were tested under the uncued paradigm to evaluate the chronic effects of cues. The number of strides was significantly reduced only in the patients that underwent cued rehabilitation. This finding represents an important goal in the rehabilitation of PD gait, typically characterized by a tendency to an increase in the number of steps. It is known that in PD patients the activity of the internal rhythm pacemaker is dysfunctional and therefore we speculate that the observed reduction in the number of strides was promoted by the pacing effect of the external cues adopted [1–7]. Interestingly the reduction in the number of strides showed a differential pattern in the two cued groups, possibly related to different mechanisms of pacing: in the Acoustic Group it was associated with an elongation in stride length, while in the Visual Group it was associated with a redistribution of the stance-swing phase of gait. It is tempting to speculate that auditory cues, once internalized with the 4-week training, are capable of providing an automatic (subcortical?) rhythm that facilitates all components and movements of gait, probably including also arms swing (although we do not have data to substantiate this speculation at this time), leading to an increased length of steps. Conversely, visual cues, with the indication to calibrate the step on a specific and steady length (the distance between tapes), may act through a less automatic, more “corticalized,” modality of training that leads to an increased attention of the patient during the swing phase for hitting the target distance.

It is important to underline that in all the three groups there was a significant increase in gait speed, without any statistical differences between groups at T1. Speed of gait represents one of the most comprehensive features of gait in Parkinson's disease which may become in certain cases an independent indicator of disease severity [21]. Our finding suggests that gait speed is not influenced by cues, being rather the effect of the multimodal exercise modalities proposed within our rehabilitative programme.

Despite the chronic effect observed at T1, we could not detect any retention after 3 months in none of the groups. This feature probably results as a combination of the progression of neurodegeneration, typical of PD, with the well-known deficit of implicit learning in PD subjects [9]. It is important however to note that our patients received precise indications to stop using cues after discharge. In the real life setting, it is conceivable that retention could be promoted by a long-term, less intensive rehabilitation with cues at home. Several studies have shown the feasibility of adopting cued gait training at home to suggest that cued training at home may actually prolong the effectiveness of inpatient treatment [33, 49, 50]. To the best of our knowledge no study has evaluated this possibility and future investigations are needed to verify its impact and feasibility.

In conclusion, our study further supports the usefulness of rehabilitation in improving gait disorders in PD. The selective impact of different kinds of cues on gait parameters suggests the usefulness of evaluating individually the gait pattern of the patients with gait analysis and testing their performance with different type of cues before the beginning of the rehabilitation programme, in order to optimize efficacy. The tendency to lose effects over months underlines once more the need for a continuative and multidisciplinary approach, characterized by serial visits and repeated rehabilitation cycles over the years.

## Figures and Tables

**Table 1:** Baseline parameters.

	Acoustic cues	Visual cues	Controls
Number of subjects	11	11	24
Age (years, m ± sd.)	78.1 ± 6.1	73.2 ± 6.9	72.1 ± 7.3
Sex (F/M)	4/7	6/5	12/12
Disease duration (years, m ± sd.)	10.0 ± 3.1	9.0 ± 2.4	10.5 ± 5.2
Patients with freezing (%)	21.2%	20.6%	22.1%
UPDRS-III	32.1 ± 9.8	29.1 ± 7.9	32.8 ± 10.8
FIM score	102.0 ± 10.2	105.8 ± 11.5	101.9 ± 19.2
Number of strides (m ± sd.)	7.2 ± 3.3	6.8 ± 2.5	7.0 ± 4.1
Stride duration (ms)	1250.5 ± 317.2	1362.9 ± 216.6	1336.7 ± 247.9
Stride length (cm)	83.5 ± 25.7	84.8 ± 19.2	86.3 ± 20.5
Stance (% of stride)	73.8 ± 7.5	71.3 ± 3.5	69.5 ± 6.0
Swing (% of stride)	26.2 ± 7.5	28.7 ± 3.5	30.5 ± 6.0
Speed (m/s)	0.63 ± 0.22	0.62 ± 0.1	0.64 ± 0.2

**Table 2:** Acute effects of acoustic cueing: comparison of gait with and without cue conditioning. Data are expressed as mean ± sd. The right column reports the *p* values for group comparison.

	Walking without cue conditioning	Walking with cue conditioning	*p* value
Number of strides	7.2 ± 3.3	7.3 ± 2.5	NS
Stride duration (ms)	1250.5 ± 317.2	1374.8 ± 381.0	<0.05
Stride length (cm)	83.5 ± 25.7	102.1 ± 31.6	<0.05
Stance (% of stride)	73.8 ± 7.5	75.5 ± 4.6	NS
Swing (% of stride)	26.2 ± 7.5	24.5 ± 4.6	NS
Speed (m/s)	0.63 ± 0.22	0.69 ± 0.32	NS

**Table 3:** Acute effects of visual cueing: comparison of gait with and without cue conditioning. Data are expressed as mean ± sd. The right column reports the *p* values for group comparison.

	Walking without cue conditioning	Walking with cue conditioning	*p* value
Number of strides	6.8 ± 2.5	4.5 ± 1.3	<0.05
Stride duration (ms)	1362.9 ± 216.6	1456.7 ± 270.1	NS
Stride length (cm)	84.8 ± 19.2	89.3 ± 12.0	NS
Stance (% of stride)	71.3 ± 3.5	65.5 ± 2.2	<0.05
Swing (% of stride)	28.7 ± 3.5	34.5 ± 2.2	<0.05
Speed (m/s)	0.62 ± 0.1	0.55 ± 0.1	<0.05

**Table 4:** Effect of acoustic cues on gait parameters: kinematic analysis of gait was performed in uncued conditions at baseline (T0), at the end of the 4-week rehabilitation period (T1), and at a 3-month follow-up (T2). Data are expressed as mean ± sd.

	T0	T1	T2	*p* value T1 versus T0	*p* value T2 versus T0
Number of strides	7.2 ± 3.3	6.2 ± 1.7	7.0 ± 4.3	<0.05	NS
Stride duration (ms)	1250.5 ± 317.2	1246 ± 263.4	1292.5 ± 214.2	NS	NS
Stride length (cm)	83.5 ± 25.7	106.7 ± 10.7	91.5 ± 11.7	<0.05	NS
Stance (% of stride)	73.8 ± 7.5	70.2 ± 3.1	74.5 ± 7.0	NS	NS
Swing (% of stride)	25.5 ± 6.9	28.5 ± 4.3	24.9 ± 8.9	NS	NS
Speed (m/s)	0.63 ± 0.22	0.77 ± 0.3	0.68 ± 0.32	<0.05	NS

**Table 5:** Effect of visual cues on gait parameters: kinematic analysis of gait was performed in uncued conditions at baseline (T0), at the end of the 4-week rehabilitation period (T1), and at a 3-month follow-up (T2). Data are expressed as mean ± sd.

	T0	T1	T2	*p* value T1 versus T0	*p* value T2 versus T0
Number of strides	6.8 ± 2.5	5.2 ± 1.0	7.1 ± 3.2	<0.05	NS
Stride duration (ms)	1362.9 ± 216.6	1332.9 ± 263.1	1384.1 ± 196.1	NS	NS
Stride length (cm)	84.8 ± 19.2	94.0 ± 29.5	84.1 ± 17.0	NS	NS
Stance (% of stride)	71.3 ± 3.5	62.6 ± 4.0	70.4 ± 4.5	<0.05	NS
Swing (% of stride)	27.6 ± 3.5	36.6 ± 3.5	29.1 ± 4.6	<0.05	NS
Speed (m/s)	0.62 ± 0.1	0.71 ± 0.2	0.65 ± 0.6	<0.05	NS

**Table 6:** Effect of gait training without cues on gait parameters: kinematic analysis of gait was performed at baseline (T0), at the end of the 4-week rehabilitation period (T1), and at a 3-month follow-up (T2). Data are expressed as mean ± sd.

	T0	T1	T2	*p* value T1 versus T0	*p* value T2 versus T0
Number of strides	7.0 ± 4.1	6.8 ± 3.5	7.4 ± 2.1	NS	NS
Stride duration (ms)	1336.7 ± 247.9	1351.8 ± 267.7	1301.7 ± 254.1	NS	NS
Stride length (cm)	86.3 ± 20.5	103.9 ± 20.7	93.3 ± 25.6	<0.05	NS
Stance (% of stride)	69.5 ± 6.0	68.8 ± 6.8	67.3 ± 5.1	NS	NS
Swing (% of stride)	30.2 ± 6.0	31.1 ± 6.7	31.5 ± 4.4	NS	NS
Speed (m/s)	0.64 ± 0.2	0.74 ± 0.3	0.66 ± 0.7	<0.05	NS

**Table 7:** Scores at UPDRS-III and FIM at baseline and at follow-ups.

		T0	T1	T2	*p* value T0 versus T1	*p* value T0 versus T2
UPDRS-III	Acoustic cues	32.1 ± 9.8	24.1 ± 9.3	31.6 ± 8.7	<0.05	NS
	Visual cues	29.1 ± 7.9	22.0 ± 4.6	28.8 ± 8.3	<0.05	NS
	Controls	32.8 ± 10.8	27.8 ± 6.3	30.4 ± 8.5	<0.05	NS

FIM	Acoustic cues	102.0 ± 10.2	111.7 ± 9.8	103.1 ± 11.3	<0.05	NS
	Visual cues	105.8 ± 11.5	111.5 ± 11.2	104.3 ± 10.6	<0.05	NS
	Controls	101.9 ± 19.2	107.7 ± 14.7	102.2 ± 15.4	<0.05	NS
